# Semisynthesis of Betaxanthins from Purified Betacyanin of *Opuntia dillenii* sp.: Color Stability and Antiradical Capacity

**DOI:** 10.3390/molecules29092116

**Published:** 2024-05-03

**Authors:** Silvia Cruz, Neyder Checa, Hugo Tovar, María Jesús Cejudo-Bastante, Francisco J. Heredia, Nelson Hurtado

**Affiliations:** 1 Grupo de Investigación en Productos de Importancia Biológica (GIPIB), Universidad de Nariño, San Juan de Pasto, Nariño 1175, Colombia; silvcruz@udenar.edu.co (S.C.); neyder013@gmail.com (N.C.); hugotovartorres@gmail.com (H.T.); nhurtado@unal.edu.co (N.H.); 2 Food Colour and Quality Laboratory, Área de Nutrición y Bromatología, Facultad de Farmacia, Universidad de Sevilla, 41012 Sevilla, Spain; heredia@us.es

**Keywords:** *Opuntia dillenii*, betaxanthins, semisynthesis, color stability, antioxidant capacity

## Abstract

The availability of pure individual betalains in sufficient quantities which permit deeper understanding is still a challenge. This study investigates the high-yielding semisynthesis of betaxanthins using betalamic acid from a natural source (*Opuntia dillenii*), followed by condensation with ʟ−amino acids and further purification. Moreover, the color stability of the four synthesized individual betaxanthins, namely proline (ʟ−ProBX), alanine (ʟ−AlaBX), leucine (ʟ−LeuBX), and phenylalanine (ʟ−PheBX) betaxanthins, was investigated at different pHs. Their relative contribution to free radical scavenging was also scrutinized by TEAC and DPPH. ʟ−AlaBX and ʟ−LeuBx showed a significantly (*p* < 0.05) higher antioxidant activity, whereas ʟ−ProBX was the most resistant to the hydrolysis of betaxanthin and hence the least susceptible to color change. The color stability was strongly influenced by pH, with the color of ʟ−ProBX, ʟ−LeuBX, and ʟ−AlaBX at pH 6 being more stable, probably due to the easier hydrolysis under acid conditions. The semisynthesis and purification allowed us to have available remarkable quantities of pure individual betaxanthins of *Opuntia dillenii* for the first time, and to establish their color properties and antioxidant capacity. This study could be a step forward in the development of the best natural food colorant formulation, based on the betalain structure, which is of special interest in food technology.

## 1. Introduction

Bioactive compounds, specifically pigments such as anthocyanins, betalains, and carotenes, isolated from natural sources, have demonstrated significant potential as food colorants and in preventing free radical damage as a food component and antioxidant agent in the pharmaceutical and cosmetic industry [1]. Additionally, drugs derived from plants containing these compounds play a crucial role in the prevention and treatment of human diseases [2,3,4]. Among these compounds, betalains are characterized by the presence of betalamic acid (4-(2-oxoethylidene)-1,2,3,4-tetrahydropyridine-2,6-dicarboxylic acid) as a structural unit, and their classification depends on the type of residue that is condensed with them. If they are amines or amino acids, they are referred to as betaxanthins, displaying a yellow-orange color. Alternatively, they are called betacyanins if they are condensed with cyclo-DOPA and its glycosylated derivatives, exhibiting a violet-purple color [5].

The fruit of *Opuntia dillenii*, a cactus plant, is typically found in tropical and subtropical regions, primarily in desert or semi-desert areas. In Colombia, the species is mainly located in the dry regions of the Santander, Nariño, and la Guajira departments [6]. The pigments found in this type of cactus belong to the betalain class [2,6]. According to a recent study published by our research group [6], the betalain extract derived from *Opuntia dillenii* sp. is a rich source of antioxidants. It contains a betacyanin content of 16.63 mg betanin/100 g fresh fruit and a betaxanthin content of 7.55 mg indicaxanthin/100 g fresh fruit, containing major betacyanins such as 17-decarboxybetanin and 17-decarboxyisobetanin, 6′-*O*-sinapoyl-*O*-gomphrenin and 6′-*O*-sinapoyl-*O*-isogomphrenine, and 2′-*O*-apiosyl-4-*O*-phyllocactin and 5″-*O*-*E*-sinapoyl-2′-apiosyl-phyllocactin, as well as betaxanthins (tryptophan, proline- and tyrosine-betaxanthins (portulacaxanthin II), and some polyphenolic compounds, such as isoramnethin-3-glucuronide and quercetin-3-*O*-glucoside.

Betalains are highly significant not only for their coloring abilities but also for their nutraceutical potential and biological activity. Several studies have reported on their anti-inflammatory, antitumor, neuroprotective, hepatoprotective, and hypotensive properties [3,5], as well as their antioxidant activity [3,4,5,6,7,8,9,10,11]. These studies indicate that extracts rich in betalains generally exhibit higher antioxidant activity compared to commonly used antioxidants such as ascorbic acid, catechin, and trolox [7,11,12]. Also, recent research has examined the potential applications of these fruit pigments in the food and cosmetic industries [2,13]. Moreover, betalains offer an advantage for the food industry as they are more water-soluble and have a coloring capacity three times greater than carotenes and anthocyanins. Additionally, they demonstrate greater stability at a pH level between 3 and 5, making them well-suited dye options for neutral or low-acid foods [14,15]. Therefore, due to consumers’ increasing demands for healthy and natural foods in both manufacturing and consumption, betalains are compounds of special interest for the food industry and deserve a deeper knowledge of their behavior.

The majority of the biological activities and color characteristics are reported with plant extracts that have undergone limited pigment purification [6,12,16,17]. While these studies are helpful for identifying potential activities, it is imperative to have available individual and pure betalains to evaluate the described effects. Nonetheless, the limited presence and low concentration of these pigments in plants, together with betalains, make them arduous to isolate from crude extracts and have a low yield [3,8,18,19], making their purification challenging. An alternative approach would be to synthesize these compounds in the laboratory. However, obtaining them by total synthesis is also challenging due to the high toxicity of the reagents, the low yields, and the numerous synthesis steps involved [20,21]. Consequently, Gandía-Herrero’s group [12,21] proposed semisynthesis protocols, based on fast-flow Q-Sepharose resin, to obtain betalamic acid through the hydrolysis of red beet betanin. Despite enabling the production of semisynthetic products, they still reported low yields or required several inefficient purification steps [22,23,24,25]. Biotechnological procedures for acquiring betalains in microbial reactors have been recently published [19], but they can be costly and time-consuming [19]. For obtaining betaxanthins specifically, there are limited reports detailing their synthesis and, unfortunately, the use of the extracted betalamic acid from crude betalain extracts can negatively impact betaxanthins’ performance and purification processes. Therefore, this study introduces the semisynthesis of betaxanthins, utilizing betacyanin fractions obtained from *Opuntia dillenii* fruit as a starting point, and using a less-polar resin based on a counterion, Cl^−^, allowing interactions via the quaternary amino groups and vinyl groups to occur. Compared to Gandia-Herrero [21], the present methodology permits the whole raw material (*Opuntia dillenii*) to be used and the entire synthesis mixture to be purified in a single step, rather than using a fast-flow chromatography system with a Q-Sepharose column. Thus, this fact allowed for a longer contact time between the synthesis mixture and the IRA-402 resin, removing the linked molecules by increasing the ionic strength through elution with Cl^−^. Betalamic acid, obtained by betacyanin hydrolysis, and different ʟ−amino acids participate in in situ condensing reactions to synthesize the respective betaxanthins. Further purification processes were also undertaken to achieve high-purity betaxanthins. This work presents the first report on the synthesis of high-yield betaxanthins from purified betacyanins of *Opuntia dilleni*. In addition, the correlation between the structural make-up and antiradical capacity (ABTS and DPPH) and their color properties depending on pH over time were scrutinized, which could be of special interest in the natural food colorant industry.

## 2. Results and Discussion

### 2.1. Isolation of the Purified Extract from the Fruit of Opuntia Dilleni and Obtaining Betalamic Acid

**Figure 1:**
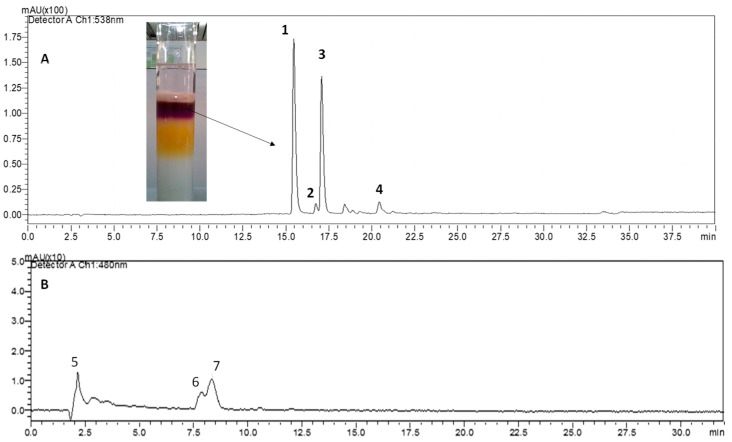
HPLC–DAD chromatogram: (**A**) Purified magenta fraction, 538 nm (PE3), and (**B**) purified yellow fraction, 480 nm. (**1**) Betanin, (**2**) 17-decarboxy-betanin, (**3**) isobetanin, (**4**) 17-descarboxy-isobetanin, (**5**) tryptophan-betaxanthin, (**6**) tyrosine-betaxanthin, (**7**) proline–betaxanthin.

The synthesis of betaxanthins utilizes betalamic acid (BA) as the starting compound, which is the structural unit of betalains. BA is typically produced by hydrolyzing crude extracts of betalains [23,24]. Our work involved isolating the crude extract (CE) (228 g) and obtaining a purified extract (PE1) that was free of mucilage and proteins. Subsequently, an extract free of colorless polyphenols (PE2) was obtained, from which the betacyanins (PE3, magenta color) and betaxanthins (yellow color) [6] were derived (Figure 1).

**Figure 2:**
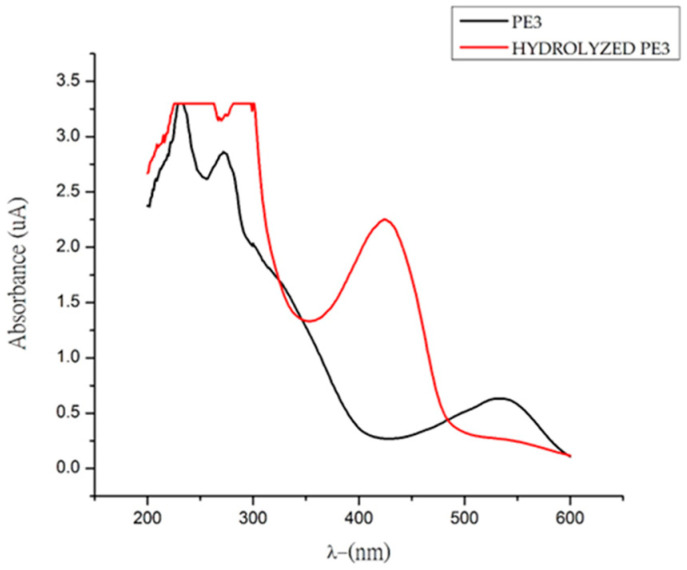
UV–Vis spectrum of the PE3 fraction before and after hydrolysis.

Spectrophotometric analysis revealed that the fruit (CE) contains 16.33 ± 0.3 mg betanin/100 g of fresh fruit and a betaxanthin content of 7.15 ± 0.1 mg indicaxanthin/100 g of fresh fruit. BA was obtained by hydrolysis of PE3; to achieve this, 10 mL of PE3 with a concentration of 11.5 mg betanin/L, which is equivalent to 2.1 × 10^−4^ mmol betanin, was used. Figure 2 shows the hydrolysis of PE3 and the formation of BA. The presence of a maximum at 424 nm indicates the formation of light–yellow BA.

This betanin, after hydrolysis, should theoretically produce 0.0443 mg of BA. In order to quantify the hydrolysis, the generated BA was purified using Amberlite IRA-402 resin, and the purified extract was immediately quantified by UV–Vis. The photometric measurement (ε = 27,000 L/mol cm) showed a final concentration of 4.68 mg BA/L (0.0441 mg of BA), indicating a percentage of hydrolysis close to 100% under the given methodological conditions.

### 2.2. Semisynthesis of ʟ−Amino Acid-Betaxanthins

**Figure 3:**
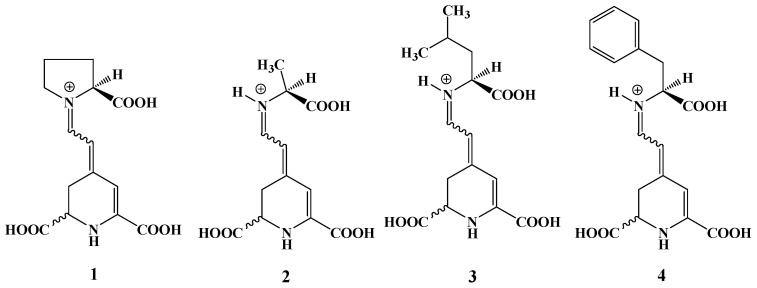
Structures of semisynthetic ʟ−amino acid-betaxanthins: (**1**) ʟ−ProBX, (**2**) ʟ−AlaBX, (**3**) ʟ−LeuBX, (**4**) ʟ−PheBX.

Due to previous publications [20,24] in which low yields were obtained when purified BA was reacted with amino acids, an in situ reaction between the two components was carried out in this work. Four betaxanthins of ʟ−amino acid origin were synthesized, namely ʟ−proline betaxanthin (ʟ−ProBX), ʟ−alanine betaxanthin (ʟ−AlaBX), ʟ−leucine betaxanthin (ʟ−LeuBX), and ʟ−phenylalanine betaxanthin (ʟ−PheBX) (Figure 3). In in situ synthesis, excess amino acids shift the equilibrium towards betaxanthin formation, thus avoiding condensation with cyclo−Dopa and reducing the number of synthesis steps. The condensation of BA with the amino acid was carried out in a single step without prior purification of the BA [21].

Finally, in order to purify the betaxanthins, it was essential to use Amberlite IRA-402. The Amberlite IRA-402 ion-exchange resin was utilized as a microreticular gel with quaternary ammonium groups as an exchange group. This is because the carboxylate groups (–COO^−^) of the betaxanthins can replace the exchangeable chloride anions of the resin, compensating for the positive charge of the quaternary ammonium groups. Unbound substances are easily removed by washing with water [22], and the betaxanthins bound to the resin are finally eluted with NaCl due to the increase in ionic strength [26].

**Figure 4:**
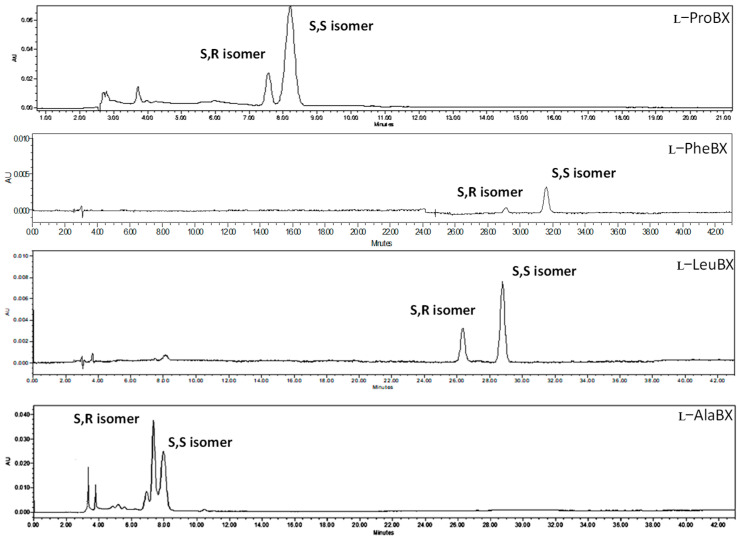
HPLC–DAD chromatogram of the synthesized betaxanthins, including the S,S (higher retention) and S,R (lower retention) diastereoisomers.

Figure 4 shows the HPLC−DAD chromatogram of the synthesized betaxanthins. The analysis of abundance data for the diastereoisomers indicated a higher abundance for the S,S isomer in all cases. This is in accordance with previous findings by other researchers [20,21]. In addition, as the BA molecule has S and R isomers in its structure, the synthesis process generated S,S and S,R diastereoisomers.

Table 1 shows the ratio, yields, and % area of the diastereoisomers. In the case of ʟ−ProBX, the S,S diastereoisomer represents 66.4% of the total area, while S,R represents 33.6%. This betaxanthin is present in *Opuntia dillenii* fruit [6] and could serve as a standard to identify this compound in other plant materials.

To calculate the reaction yields, in the case of ʟ−ProBX, 7 mL of PE3 (which is equivalent to 1.17 × 10^−3^ mmol BA) was mixed with 0.819 mmol ʟ−proline, which theoretically should yield 1.17 × 10^−3^ mmol ʟ−ProBX (0.361 mg). The final concentration was 16.22 mg ʟ−ProBX/L (8.92 × 10^−4^ mmol ʟ−ProBX, 0.275 mg), with a yield of 76.2% (mg ʟ−ProBX/100 mg ʟ−ProBX theoretical). The same procedure was followed for the synthesis of the other betaxanthins (Table 1). Despite the lack of reports using this type of synthesis from purified betacyanin extracts, some groups have published lower yields than those obtained in this study [20,24,25].

### 2.3. Tentative Characterization of Betaxanthins by UV-Vis and HPLC-ESI-MS

Table 2 and Figure S1 present the *m*/*z* ratio of the pseudomolecular ion in positive [M + H]^+^ mode. The ʟ−ProBX compound exhibited an *m*/*z* of 310.02547 [M + H]^+^ units, which was confirmed by the presence of an absorption peak at λ_max_ 484 nm due to the absorption generated by the π→π* transitions characteristic of betaxanthins.

The pseudomolecular ion for the ʟ−AlaBX molecule was observed at *m*/*z* 284.16047 [M + H]^+^. For ʟ−LeuBX, the pseudomolecular ion was observed at *m*/*z* 326.80460 [M + H]^+^, and for ʟ−PheBX, the pseudomolecular ion was observed at *m*/*z* 360.13 [M + H]^+^ in positive mode (Table 2). Comparing the retention times with those published in the literature [18,19,26], it is observed that they follow the same trend, i.e., the S,S diastereoisomers retained more than the S,R ones. These data, together with their λ_max_ (Table 1) and mass values, are similar to those reported in the literature [20,21], confirming the synthesis of these compounds.

### 2.4. Determination of the Antioxidant Capacity

Due to the complexity of oxidative reactions, a single assay may not be sufficient to predict all oxidative details in molecules. Therefore, antioxidant assays based on electron transfer (such as TEAC and DPPH) were conducted to estimate the antioxidant potential of the synthesized betaxanthins. The mechanism is based on the electron transfer capacity of betaxanthins to inhibit ABTS^•+^ and DPPH^•^ radicals. As different studies have found that the antioxidant capacity depends on the pH of the surrounding medium, in this study, measurements were carried out at pH 7 (Table 3). The TEAC value of ascorbic acid (1.24 ± 0.01 mmol trolox/mmol BX), the positive control, agreed with that used by other authors [27].

Table 3 shows the ability of the synthesized molecules and betalamic acid to reduce ABTS^•+^ and DPPH^•^ radicals. According to data analysis using Fisher’s least significant difference (LSD) procedure with a confidence level of 95% and a statistical significance of *p* < 0.05, betalamic acid exhibits superior antioxidant properties compared to ascorbic acid.

The antiradical capacity followed the following order: ʟ−AlaBX > ʟ−LeuXx > ʟ−PheBX, ʟ−ProBX. The analysis of the synthesized betaxanthins indicates that the ʟ−AlaBX molecule was more effective in capturing the free radicals ABTS^•+^ and DPPH^•^ than ʟ−ProBX, ʟ−LeuBX, and ʟ−PheBX (Table 3). The pigments selected for synthesis in this study exhibit amino acids with structural diversity that reacted with the aldehyde group of betalamic acid through a Schiff condensation reaction. The pigment ʟ−PheBX contains a phenyl group in its structure, while ʟ−AlaBX and ʟ−LeuBX have a linear chain to the carboxylic acid. Furthermore, ʟ−ProBX has a positively charged iminium group within its structure, as depicted in Figure 3. Therefore, it seemed that molecules with a linear chain to the carboxylic acid show the highest antiradical capacity (Table 3).

Likewise, significant differences in TEAC’s and DPPH’s antiradical capacity were observed between ʟ−ProBX, which has a lower antioxidant capacity, and ʟ−AlaBX, ʟ−LeuBX, and betalamic acid (Table 3). The difference between the values could be associated with the findings of Esteves [24], Fernando [9], and Gandía-Herrero [28], who suggest that the presence of the positively charged iminium group of the secondary amine causes a decrease in antioxidant capacity. This behavior demonstrates how the structural characteristics of the molecule affect its capacity to eliminate free radicals. In the case of ʟ−Phe-BX, which has an antiradical activity comparable to ʟ−ProBX, the low antiradical activity may be due to the fact that the aromatic ring of this molecule does not participate in the resonance delocalization of the 1,7-diazaheptamethinium system [25,28]. The lower antiradical activity of ʟ−ProBX can be attributed to the presence of the imino group of the secondary amine, which stabilizes the molecule. In the case of ʟ−PheBX, its low activity may be due to the fact that the aromatic ring does not participate in resonance delocalization.

These findings are in accordance with previous research [25,28] and help in understanding antiradical mechanisms. The different structural characteristics of the synthesized betaxanthins have been studied in previous research on betaxanthins [28,29].

### 2.5. Stability Evaluation of Semisynthetic ʟ−Amino Acid-Betaxanthins by Tristimulus Colorimetry

This study examines how the structure of betaxanthins, produced by creating the aldimine bond between betalamic acid and four chosen amino acids for semisynthesis, influences the color and storage stability at pH 4 and pH 6. The four synthesized betaxanthin solutions were kept for eight days at room temperature (18 °C) in the dark and in the presence of oxygen at pH 4 and pH 6.

Based on the CIELAB parameters (L*, a*, b*, C*_ab_, h_ab_) (Table 4), the color stability of betaxanthins is dependent on both pH and structure. The aqueous solutions of the betaxanthins (5 × 10^−5^ M) prepared early (t = 0) showed the highest chroma value (C*_ab_) at the highest pH value (pH 6), indicating a greater intensity of color (higher color purity). However, at this pH, the solutions tended to be lighter (higher L*). The molecule ʟ−AlaBX exhibited the highest C*_ab_ value (51.0, pH 6), that is, it showed a greater color intensity (greater color purity), while ʟ−PheBX exhibited the lowest chroma value (16.4, pH 4). Furthermore, at pH 4, all betaxanthin solutions displayed yellow-green tonalities ranging from 94.5° to 85.6°, while at pH 6, the hues were slightly more yellow, ranging from 91.4° to 90.3°.

**Figure 5:**
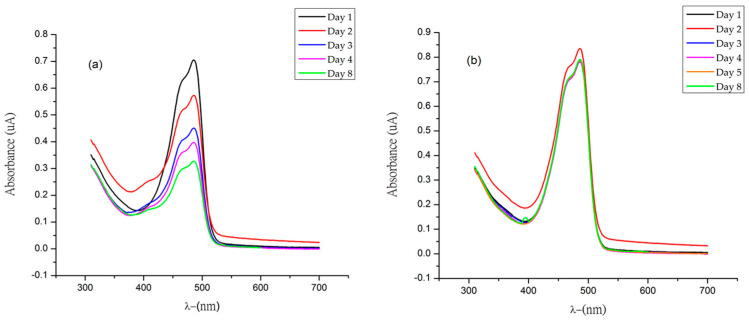
Evolution over time of the absorbance and UV–Vis spectrum of the most stable betaxanthin, ʟ−ProBX: (**a**) UV-Vis pH 4, (**b**) UV-Vis pH 6.

Thus, it is clear that pH has an impact on the CIELAB parameters of the initial betaxanthin solutions. However, the colorimetric characteristics (L*, C*_ab_, h_ab_) and color differences (∆E*_ab_) showed different patterns of evolution over time with respect to pH. After the storage period at pH 6, the solutions of betaxanthins ʟ−AlaBX, ʟ−LeuBX, and ʟ−ProBX experienced the smallest changes in lightness, chroma, and hue (∆L*, ∆C*_ab_, and ∆h_ab_) (Table 3). In contrast, ʟ−PheBX showed drastic changes in L* and C*_ab_ at the same pH value (∆L* = 42.4 and ∆C*_ab_ = 30.8). This indicates that when stored at pH 4, the chroma and luminosity present less variation during this time interval (Table 4). The colorimetric parameters of the aqueous solution of ʟ−ProBX experienced the least changes at pH 6, while at pH 4, the ʟ−PheBX solution had the least variation in the parameters L* and C*_ab_. It is important to note that these observations are objective and based on the experimental data. Table 4 shows that the color changes (ΔE*_ab_) over time differed among the four betaxanthin solutions stored at pH 4 and pH 6. Overall, greater color differences were observed for all solutions at pH 4 compared to those stored at pH 6, except for the ʟ−PheBX solution. The greater color degradation (less stability) at pH 4 may be attributed to degradation by acid hydrolysis, similar to what was observed by Herbach when studying the color stability of betalain extracts [30]. Hydrolysis results in the cleavage of the aldimino bond, isomerization, decarboxylation, and dehydrogenation at the chiral carbon, or decarboxylation of betalamic acid. The present study observed a gradual loss of yellow color in the case of betaxanthins. It has been confirmed that a ΔE*_ab_ greater than two CIELAB units indicates color differences that are appreciable to the human eye [31]. The aqueous solutions of betaxanthins ʟ−AlaBX, ʟ−LeuBX, and ʟ−PheBX displayed noticeable color differences when stored at pH 4 and pH 6 (∆E*_ab_ > 2); however, the changes in ∆E*_ab_ were less drastic at pH 6. ʟ−ProBX showed minimal color changes at pH 6 (∆E*_ab_ = 0.64), making it the most stable synthesized betaxanthin under these storage conditions, with the least hydrolytic degradation (Figure 5). This behavior suggests that the previously observed reduction in antioxidant capacity for ʟ−Pro-BX may be attributed to the greater stability of this particular molecule. The presence of the cyclopentyl ring effectively stabilizes the iminium cation, resulting in greater stabilization and decreased hydrolysis. The chemical structure clearly affects color stability during storage at both pH levels.

As previously mentioned, the ʟ−PheBX solution displayed the smallest color differences at pH 4, although they were visually noticeable (ΔE*_ab_ = 10.6). This analysis agrees with the previously observed variations in chroma, lightness, and hue in the solutions of ʟ−ProBX (which exhibits higher color stability at pH 6) and ʟ−PheBX (which exhibits higher color stability at pH 4).

The observed discrepancy may be attributed to the higher acid dissociation constant (Ka = 6.7 × 10^−3^) of the amino acid condensed to betalamic acid to form ʟ−PheBX, in this case, phenylalanine, compared to other amino acids such as alanine (Ka = 4.89 × 10^−3^) [31]. Therefore, it can be assumed that it is hydrolyzed more easily in a less-acidic medium (pH 6). The research conducted by Abu-Eittah [32] found that phenylalanine has a lower dipole moment (μ = 2.2909 D) compared to alanine (μ = 2.5622 D). This suggests that the polarization of the benzene ring is in the opposite direction to that of the –COOH group, which explains why phenylalanine is a stronger acid than alanine.

**Figure 6:**
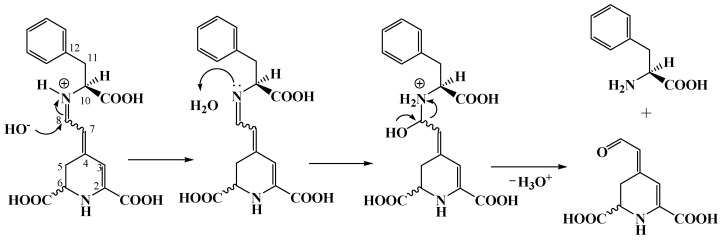
Hydrolysis mechanism in basic medium of ʟ−PheBX.

This work only used phenylalanine as the aromatic amino acid in the synthesis of betaxanthins. The ʟ−PheBX solution was found to be the least resistant to color changes (ΔE*_ab_ = 52.4) at a pH value of 6 (Table 4). Moreover, the phenylalanine has a slightly negative charge on the benzene ring, indicating its potential to function as an electron donor and acceptor [32], which can lead to faster hydrolysis (Figure 6) and less stabilization of the iminium bond by the benzyl group compared to other amino acids attached to the semisynthetic betaxanthins under examination, an aspect that is in accordance with what was published by Abu-Eittah [32]. Imine hydrolysis occurs easily in both acidic and basic media and has been extensively analyzed using kinetic methods. The reaction mechanism is dependent on the composition of the reactant molecule and the pH of the solution. Under mildly acidic conditions, the imine bond breaks as the hydroxyl group adds to the C=N bond; then, the amino group of betaxanthin receives a proton from a water molecule, causing the elimination of the amino acid (Figure 6).

In summary, after the storage period at both pH values, appreciable differences were observed in the color of the solutions of ʟ−AlaBX, ʟ−LeuBX, and ʟ−PheBX; on the contrary, ʟ−ProBX is more resistant to changes in pH. Color differences at pH 6 were not perceptible to the human eye, possibly being one of the compounds that contributes the most to the stability of the crude extract of Opuntia dillenii. These findings on color stability in relation to pH align with Cejudo-Bastante [17], who noted that the hue in crude extracts of Opuntia dillenii fruit is more stable in less acidic pH levels. These results are consistent with the data presented in the work of Wu [33], which demonstrated that storage at room temperature (23–27 °C) of Bougainvillea extracts rich in betaxanthins maintains the yellow color. At pH values between 5 and 7, the extracts retain their color, while at pH values less than 5, they experience a loss of color.

## 3. Materials and Methods

### 3.1. Reagents and Solvents

All reagents and solvents (ethanol, methanol, glacial acetyl acid, sodium hydroxide, formic acid, ammonium hydroxide, triethylamine, ʟ−alanine, ʟ−proline, ʟ−leucine, ʟ−phenylalanine, 2,2-diphenyl-1-picrylhydrazine (DPPH), 2,2′-azino-bis-(3-ethylbenzothiazolin)-6-sulfonic acid (ABTS), potassium persulfate, gallic acid, Amberlite XAD-7, Amberlite IRA-402, and silica functionalized with C18 groups) were purchased from Sigma-Aldrich (St. Louis, MO, USA).

### 3.2. Plant Material

**Figure 7:**
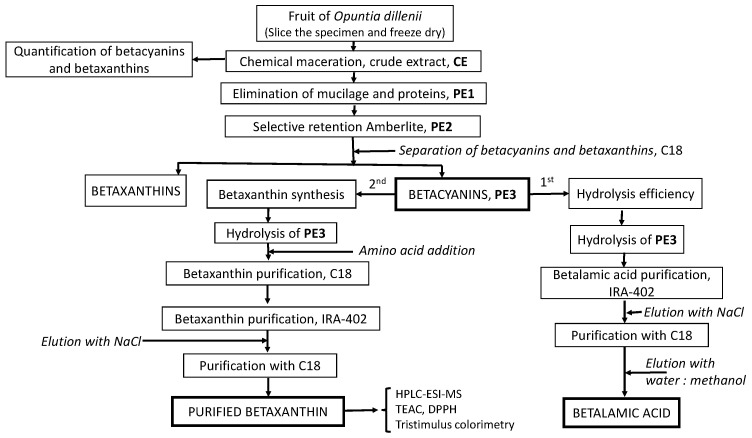
Methodological scheme of the synthesis and purification of betalains.

The samples were collected from the village of Chachagui (Nariño, Colombia), which is located approximately 25 km north of the city of San Juan de Pasto. It is located approximately at 1°24′31″ latitude N and 77°17′23″ longitude W at an altitude of 1950 m above sea level, with an average temperature of 18 °C. On 15 October 2020, the fruit was harvested, and a specimen of the plant was stored in the herbarium of the Universidad of Nariño (code number 13691). A representative set of samples of up to a weight of about 2.5 Kg was harvested. The mature fruits were collected according to visual characteristics and similar size. A simple random sampling model of ten plants was performed. After homogenization, only 1200 g of fruits was considered, with an average weight of each fruit being around 10 g. They were carefully washed and dried with paper, and the prickles were manually removed. Fruits were kept under refrigeration at 4 °C and then lyophilized until their analysis. Figure 7 shows the methodological scheme of the synthesis and purification of betalains from the fruits of *Opuntia dillenii*.

### 3.3. Preparation of Crude Extract (CE) and Purified Extract (PE1)

Initially, the crude extract (CE) was obtained according to the method described by Betancourt [6]. Briefly, 600 g of lyophilized fruits was cut into small pieces (1 cm^2^) and extracted with a 2.5 mL/g sample of methanol/water (60:40) for 24 h at 10 °C (chemical maceration). The procedure was repeated until the solvents used were colorless. Subsequently, the organic solvent was removed at 35 °C using a rotary evaporator (Heidolph, Schwabach, Germany), and the extract was re-dissolved with distilled water (1 g/mL) and then lyophilized (Labconco, MO, USA), resulting in the isolation of 228 g of crude extract (CE) [6]. Subsequently, to obtain simpler fractions and facilitate betalain purification, the CE (10 g) was first subjected to mucilage and protein separation by precipitation. Briefly, 20 mL of water and 40 mL of 96% ethanol (1:2) were added to the CE and stirred for 20 min. After centrifugation, the mucilages and proteins were separated from the liquid phase with a filter paper (Whatman No. 1 filter paper). Subsequently, the organic solvent was evaporated at 30 °C using a rotary evaporator at 70 rpm (Heidolph, Schwabach, Germany). The purified extract (PE1) was then lyophilized and kept at 5 °C until analysis (Figure 7). The procedure was carried out in triplicate.

### 3.4. Separation of Polyphenols with Ambertite XAD-7 from PE1

Selective retention of betalains and polyphenols using ion-exchange chromatography with a non-ionic polymeric absorbent Amberlite XAD-7 was undertaken. The column (20 × 2 cm) (25 g) was conditioned by passing three volumes of water. Subsequently, the sample (3 g PE1/5 mL deionized water) was introduced. Betalains were eluted from the column with three volumes of water type II (PE2), and then the betalain extract (PE2) was lyophilized (Figure 7). The colorless polyphenols, together with sugars and organic acids, from PE1 that were retained in the resin were eluted with methanol/acid (19:1) to be discarded [34].

### 3.5. Isolation of Betacyanins from PE2

Fractionation based on hydrophobicity was carried out by column chromatography. To separate betacyanins from betaxanthins, a column (20 × 2 cm) with 35 g of C18 stationary phase was first conditioned by passing, separately, three volumes of 100% methanol and then three volumes of acidic deionized water with formic acid (pH 3) [6]. Subsequently, the sample (1 g PE2/5 mL deionized water) was added to the column. Two fractions were eluted based on their color: (a) the yellow fraction (betaxanthins, less-hydrophobic compounds), using three volumes of water acidified with formic acid (pH 3), and (b) the magenta fraction (betacyanins, PE3), with four volumes of a mixture of acetone/acidified water (60:40, *v*/*v*). The betacyanin fraction was concentrated under vacuum and then lyophilized (Figure 7).

### 3.6. Quantification of Betacyanins and Betaxanthins

Spectra were recorded in triplicate within the range 360–800 nm by spectrophotometry (Merck, Spectroquant^®^ Pharo 300, Rahway, NJ, USA). Two absorbances at maxima (480 and 538 nm) were reported for the quantification of betaxanthins (yellow fraction) and betacyanins (magenta fraction), respectively. The betacyanin and betaxanthin content (B) was determined using the following equation:

[B] (mg/g) = [(Abs)(DF)(V)(MW)/(ε)(L)(W)] (1)

where Abs is the maximum absorbance value at 480 or 538 nm, DF is the dilution factor, V is the volume (mL) of the extracts, MW and ε are the molecular weight and the molar extinction coefficient of betanin (550 g/mol and 60,000 L/mol cm in H_2_O) and the values for the four synthesized betaxanthins, L is the optical path length (0.2 cm), and W is the weight of the sample (g). All analyses were performed in triplicate. For the quantification of betalamic acid, the same equation for the quantification of betalains was used, taking into account the following parameters: Abs is the absorbance at 424 nm (in H_2_O); MW = 211.17 g/mol; and ε = molar extinction coefficient (27,000 L/mol cm) [35].

### 3.7. Obtaining Betalamic Acid

Betalamic acid (BA) was obtained by basic hydrolysis from 10 mL of the purified extract containing betacyanins (PE3) in order to quantify the efficiency of hydrolysis in obtaining BA. The hydrolysis was carried out with the addition of ammonium hydroxide (NH_4_OH) to PE3 until a pH of 11.0 to 11.4 was reached [21]. BA was purified by anion-exchange chromatography, using a quaternary ammonium cross-linked anion-exchange resin IRA-402, which retains betalamic acid through ionic interactions. For that, BA was interacted with the resin for 15 min by stirring, followed by centrifugation at 5000 rpm for 5 min. After removing the supernatant and washing the resin with water to a neutral pH, the BA bound to the resin was eluted with 4 M NaCl. After the elution of the BA, and due to the presence of 4 M NaCl, a solid-phase extraction step was performed in a C18 column to remove sodium chloride salts [22]. For that, the C18 column (20 × 2 cm) was first conditioned with ethanol, followed by water pH 3.0, and neutralized with type II water (Figure 7). The sample was then added and washed with 2 L of type II water, ensuring a complete elution of the salt. Subsequently, the BA was eluted with water/methanol (19:1), confirmed by the elution of a pale-yellow color fraction and an absorbance value at 424 nm [35].

### 3.8. Semisynthesis of ʟ−Amino Acid-Betaxanthins

For the preparation of large quantities of betaxanthins by semisynthesis, Gandía-Herrero’s [21] protocol with minor changes was used. The technique involves a betalamic acid and amino acid condensation reaction, where BA was acquired from the fundamental hydrolysis of the isolated and purified betacyanins (PE3) from *Opuntia dillenii* fruit.

The synthesis of all betaxanthins started with the mixture of 7 mL PE3 (the concentration of PE3 was adjusted in each case to achieve the appropriate absorbance) hydrolyzed with NH_4_OH (achieving a pH between 11.0 and 11.4) and an excess of the corresponding amino acid (ʟ−proline, ʟ−alanine, ʟ−leucine, ʟ−phenylalanine) in a ratio of 1:700 (BA/amino acid), and continuously stirred for 20 min (Figure 7). To avoid oxidation during hydrolysis, the solution was maintained under a nitrogen atmosphere. The end of hydrolysis was evidenced by the color change from magenta to pale yellow. The monitoring was carried out by UV–Vis spectroscopy, observing the change in absorbance from λ_max_ of 536 nm (betacyanins) to 424 nm (betalamic acid) [35]. Finally, to allow the formation of aldimine bonds between BA and the ʟ−amino acid, the pH was adjusted to 5.0 with acetic acid, conditioning the solution in an ice bath when adding the acetic acid since low temperatures favor its formation [30]. The tests were carried out in triplicate.

### 3.9. Betaxanthins Purification

After semisynthesis, the solution was concentrated under reduced pressure at 35 °C to remove excess amino acids, salts, and cyclo-DOPA-glucosides. A purification protocol was then implemented to eliminate reaction by-products, as described by Cabanes [22]. Initially, the concentrated sample was passed through a C18 column (20 × 2 cm) and washed with water type II to eliminate the by-products of the reaction. Then, each semisynthetic betaxanhtin was eluted using three volumes of water acidified with formic acid (pH 3). Afterward, in order to eliminate colorless polyphenols and other remaining impurities, the semisynthetic ʟ−amino acid-betaxanthins interacted with an anion-exchange resin (Amberlite IRA-402, 20 g) while undergoing magnetic stirring for 15 min. The resultant mixture was centrifuged at 5000 rpm for 5 min. After discarding the liquid above the sediment and rinsing the resin with water until it reached a neutral pH, the resin was loaded onto a chromatography column. Subsequently, 4 M NaCl solution was passed through the column to elute the betaxanthins, followed by another wash with water type II using a C18 column in order to eliminate NaCl. Finally, the betaxanthins were eluted using a mixture of water and methanol (19:1), concentrated in a rotary evaporator, and then lyophilized for further analysis (Figure 7).

### 3.10. Analysis of Betaxanthins by UHPLC–ESI–MS

A Shimadzu UHPLC Nexera X2 attached to a Shimadzu (Tokyo, Japan) LCMS-9030 QTOF with ESI source was used. Once samples were filtered (0.45 μm nylon filter, Sigma-Aldrich, St. Louis, MO, USA), betalains were separated using a Shim pack XR–ODS C18 column (150 × 2 mm, 2.2 μm particle size), at 7500 psi maximum working pressure and maximum flow rate of 0.4 mL/min, and using 1% formic acid in water (*v*/*v*, eluent A) and a mixture of acetonitrile/water/formic acid (80:19:1) (eluent B) (Sigma-Aldrich, St. Louis, MO, USA). The volume injection was 5 μL. The chromatographic method started with 100% of A, followed by a linear gradient from 0 to 20% of B in 35 min and then a linear gradient from 20 to 100% of B in 5 min [6]. To re-establish the initial conditions, a linear gradient from 100% of B to 100% of A was used for 10 min. The identification of the individual betalains was performed in positive mode using a sweeping range of *m*/*z* 100 to 1000 u. An amount of 4.5 mL/min of nitrogen was used as the drying gas, the temperature was set at 300 °C, the nebulizer gas flow was 3 L/min, the interface temperature was 526 °C, the sampling rate was 5 µ/s, the heater gas flow was 10 L/min, and the detector voltage was 0.20 kV.

### 3.11. Determination of Antioxidant Capacity Equivalent to Trolox (TEAC)

The antioxidant capacity was measured in vitro based on the ability to scavenge the ABTS^•+^ radical, which was produced by the oxidation of 7 mM of ABTS with 2.45 mM of potassium persulfate in water. The ABTS^•+^ solution was diluted with phosphate-buffered saline (PBS) and adjusted to pH 7.0 after storage under dark conditions and room temperature for 16 h until reaching an absorbance of 0.70 ± 0.02 at 734 nm. Subsequently, 30 μL of betaxanthin was added to 3 mL of the diluted solution of ABTS^•+^. After stirring for 1 min and waiting for 6 min, the absorbance at 734 nm was spectrophotometrically measured (UV-Vis Pharo, Merck, Darmstadt, Germany) [34,36]. Results were obtained by interpolation of the absorbance in the trolox calibration curve (0.5–2.5 μM). The values of the antioxidant activity of ascorbic acid used as a positive control were in agreement with other authors (1.08 mmol trolox/mmol ascorbic acid) [27]. The results were expressed as mmol of trolox equivalent/mmol betaxanthin. Values were expressed as mean ± SD (*n* = 3).

### 3.12. Determination of Antioxidant Activity by 2,2-Diphenyl-1-Picrylhydrazyl (DPPH)

An aliquot of each sample (0.1 mL) was added to 3.9 mL of a 6.49 × 10^−5^ mol/L of DPPH in methanol [36]. To determine the percentage inhibition of the DPPH^•^ radical, the absorbance was monitored at 515 nm using a UV–Vis Spectroquant-Pharo 300 (USA) after 30 min. As a control sample, 0.1 mL of ethanol was mixed with 3.9 mL of DPPH^•^. The percentage inhibition was calculated using the following equation, where A is the absorbance at 515 nm. The calculation for the inhibition percentage is determined through the following equation:

% inhibition = (∆Abs/Abs max) × 100 (2)

where ∆Abs is the difference in absorbance of the radical cation in the absence and presence of the antioxidant at 734 nm, and Abs max is the maximum absorbance of the radical cation in the absence of the antioxidant. Values were expressed as mean ± SD (*n* = 3).

### 3.13. Colorimetric Measurements at Different pH

A Hewlett-Packard UV–vis HP8453 spectrophotometer (Palo Alto, CA, USA) was employed to develop color measurements. The visible spectrum ranging from 380 to 770 nm was consistently recorded using glass cells with 10 mm path length and distilled water as a reference medium at 2 nm intervals. To calculate the CIELAB parameters, the authentic CromaLab© software was used [37], considering the recommendations of the CIE 1964 10° Standard Observer and the standard D65 illuminant [38]. The Euclidean distance was utilized to compute color differences (ΔE*_ab_) between points in a three-dimensional space defined by L*, a*, and b*: ΔE*_ab_ = [(ΔL*)^2^ + (Δa*)^2^ + (Δb*)^2^]^1/2^ [26]. To assess color stability, semisynthetic betaxanthins (5 × 10^−5^ M) were stored in aqueous solutions for 8 d at pH 4 and 6, in a dark room, and at room temperature (18 °C). A shorter storage time (3 and 4 d) due to low absorbance values after this period was considered for some betaxanthins (ʟ−PheBX and ʟ−LeuBX, respectively). The pH alteration was achieved by using a buffer solution. The presented data were obtained in duplicate and expressed as mean ± SD.

### 3.14. Statistical Analysis

Statistical analyses were carried out using the Statgraphics Centurion 16.1.15 software (Virginia, VA, USA). The univariant variance analysis (ANOVA) using a general linear model program was applied to establish whether the mean values of the sample data differed significantly from each other. The mean values from each set of samples (*n* = 3) were compared using Fisher’s least significant difference (LSD) procedure.

## 4. Conclusions

The proposed methodology resulted in high yields in the semisynthesis of betaxanthins for the first time, offering new possibilities for betaxanthin research and the future applications of these pigments since it could be possible to have available high quantities of pure betaxanthins, which are of special interest for the food colorant industry. The identity of the molecules was verified using mass spectrometry (UHPLC–ESI–MS) and UV–Vis spectroscopy. Additionally, this study demonstrated the relationship between the chemical structure, antioxidant activity, and color stability of betaxanthins, and their relevance to changes in pH.

The presence of a secondary amine (ʟ−ProBX) and the lack of resonance delocalization (ʟ−PheBX) seem to be responsible for the low antiradical activity, whereas the linear chain to the carboxylic acid was related to the improvement in the ability to eliminate free radicals. Furthermore, the color of the synthesized betaxanthins was more stable at pH 6, particularly that of ʟ−ProBX. Therefore, although ʟ−ProBX is one of the least effective antioxidants, it was the most colorimetrically stable and could be used as a food coloring in a wider pH range.

These findings offer a range of possibilities focused on the optimization of the formulation of natural food colorants, based on the structure of their betalains, that have better antioxidant and color properties. Finally, while the combined impact of antioxidants and the effect of factors such as pH, temperature, and light on color stability cannot be ignored, our findings suggest that recognizing the role of structural characteristics in reducing power and color stability may improve the understanding of their influence on more complex extractions.

## Figures and Tables

**Table 1:** UV−Vis data, experimental and theoretical ratio, and yield of synthesized betaxanthins and abundance of each stereoisomer.

Betaxanthin	λ_max_	Ratio Experimental/Theoretical	Yield (%)	% Area (HPLC) S,S/S,R
ʟ−ProBX	484	0.275/0.361	76.2	66.4/33.6
ʟ−AlaBx	475	0.149/0.198	75.3	47.8/45.2
ʟ−LeuBX	475	0.267/0.379	70.1	72.7/27.3
ʟ−PheBX	477	0.482/0.697	69.2	86.3/13.7

**Table 2:** Molecular formula, retention times, and mass spectral values of semisynthetic betaxanthins identified by UHPLC-ESI-MS.

Betaxanthin	Molecular Formula	R_t_ (min) (S,S/S,R)	*m*/*z* [M + H]^+^
ʟ−ProBX	C_14_H_17_N_2_O_6_	8.6/7.9	310.02547
ʟ−AlaBx	C_12_H_15_N_2_O_6_	5.4/5.1	284.16047
ʟ−LeuBX	C_15_H_21_N_2_O_6_	28.8/26.4	326.80460
ʟ−PheBX	C_18_H_19_N_2_O_6_	32.0/29.5	360.12592

**Table 3:** Antioxidant capacity values by TEAC and DPPH.

Betaxanthin	TEAC (mmol Tx/mmol BX)	DPPH (% Inhibition)
ʟ−ProBX	1.17 ± 0.07 ^a^	9.85 ± 1.48 ^a^
ʟ−AlaBx	2.27 ± 0.04 ^b^	16.22 ± 1.49 ^b^
ʟ−LeuBX	1.61 ± 0.03 ^c^	12.54 ± 1.36 ^c^
ʟ−PheBX	1.27 ± 0.03 ^a^	11.32 ± 1.97 ^c^
Betalamic acid	2.15 ± 0.62 ^b^	8.73 ± 1.39 ^a^

Values are expressed as mean ± SD (*n* = 3). Values in the same column followed by different letters are significantly different by ANOVA test (*p* < 0.05).

**Table 4:** CIELAB color parameters (L*, a*, b*, C*_ab_, h_ab_) of semisynthesized betaxanthins before (BS) and after (AS) the storage period, visual color based on CIELAB color parameters, and lightness, chroma, and hue differences (∆L*, ∆C*_ab_, ∆h_ab_) and color differences (∆E*_ab_) over time.

Betaxanthins	pH	Storage	Visual Color	L*	a*	b*	C*_ab_	h_ab_	∆L*	∆C*_ab_	∆h_ab_	∆E*_ab_
ʟ−Ala-Bx	4	BS		93.8 ± 0.22	−2.1 ± 0.03	37.2 ± 0.07	37.3 ± 0.10	93.2 ± 0.37	3.4	20.6	0.35	20.9
		AS		97.2 ± 0.38	−1.0 ± 0.01	16.7 ± 0.04	16.7 ± 0.05	93.5 ± 0.28				
	6	BS		92.9 ± 0.15	−1.2 ± 0.03	50.9 ± 0.11	51.0 ± 0.13	91.4 ± 0.18	1.5	7.9	0.50	8.1
		AS		94.3 ± 0.09	−1.4 ± 0.05	43.0 ± 0.08	43.1 ± 0.07	91.8 ± 0.13				
ʟ−Leu-Bx	4	BS		94.2 ± 0.13	−1.5 ± 0.02	35.4 ± 0.07	35.5 ± 0.09	92.4 ± 0.19	2.9	25.5	5.6	25.7
		AS		97.0 ± 0.11	0.55 ± 0.00	10.0 ± 0.04	10.0 ± 0.08	86.9 ± 0.16				
	6	BS		93.1 ± 0.08	−0.7 ± 0.01	42.2 ± 0.11	42.2 ± 0.11	91.0 ± 0.13	2.0	15.3	1.1	15.4
		AS		95.1 ± 0.21	−1.0 ± 0.01	26.9 ± 0.06	26.9 ± 0.04	92.1 ± 0.11				
ʟ−Phe-Bx	4	BS		97.3 ± 0.19	−1.2 ± 0.02	16.3 ± 0.08	16.4 ± 0.05	94.4 ± 0.10	1.3	10.5	2.0	10.6
		AS		98.6 ± 0.27	−0.7 ± 0.00	5.9 ± 0.05	5.9 ± 0.04	96.3 ± 0.21				
	6	BS		92.3 ± 0.15	−0.2 ± 0.00	43.5 ± 0.09	43.5 ± 0.09	90.3 ± 0.14	42.4	30.8	1.6	52.4
		AS		49.9 ± 0.08	0.29 ± 0.01	12.7 ± 0.10	12.7 ± 0.03	88.7 ± 0.21				
ʟ−Pro-Bx	4	BS		93.6 ± 0.09	3.3 ± 0.03	43.9 ± 0.15	44.1 ± 0.09	85.7 ± 0.21	2.6	18.8	0.72	18.9
		AS		96.2 ± 0.20	1.6 ± 0.00	25.3 ± 0.07	25.3 ± 0.03	86.4 ± 0.09				
	6	BS		93.3 ± 0.16	3.7 ± 0.02	47.4 ± 0.08	47.5 ± 0.07	85.5 ± 0.07	0.27	0.44	0.46	0.64
		AS		93.6 ± 0.11	4.1 ± 0.04	47.8 ± 0.08	48.0 ± 0.08	85.1 ± 0.19				

## Data Availability

Data are contained within the article and Supplementary Materials.

## Supplementary Materials

The following supporting information can be downloaded at https://www.mdpi.com/article/10.3390/molecules29092116/s1: Figure S1: Mass spectrum of the synthesized betaxanthins.
