# Supplementary material for: Semisynthesis of Betaxanthins from Purified Betacyanin of Opuntia dillenii sp.: Color Stability and Antiradical Capacity
Source: Molecules. 2024 May 3;29(9):2116. doi: 10.3390/molecules29092116 (PMC11085281; doi:10.3390/molecules29092116)

l-LeuBx mass spectrum

<Spectrum>

Line#:1 R.Time:----(Scan#:----)
MassPeaks:1065
RawMode:Averaged 0.688-0.692(414-416) BasePeak:206.89336(21754)
BG Mode:Calc Segment 1 - Event 1

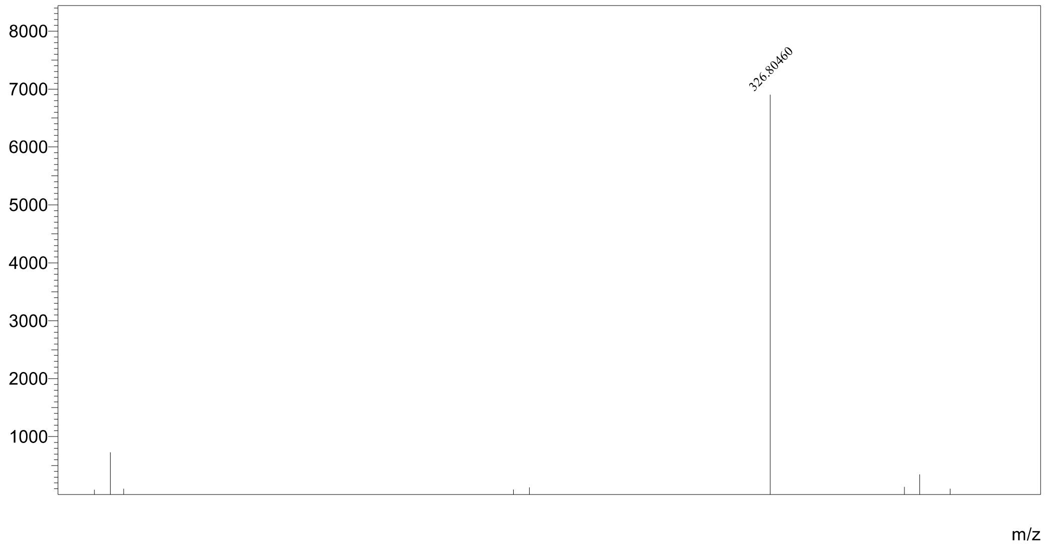

l-ProBx mass spectrum

<Spectrum>

Line#:1 R.Time:----(Scan#:----)
MassPeaks:1574
RawMode:Averaged 0.790-0.793(475-477) BasePeak:434.12433(30343)
BG Mode:Calc Segment 1 - Event 1

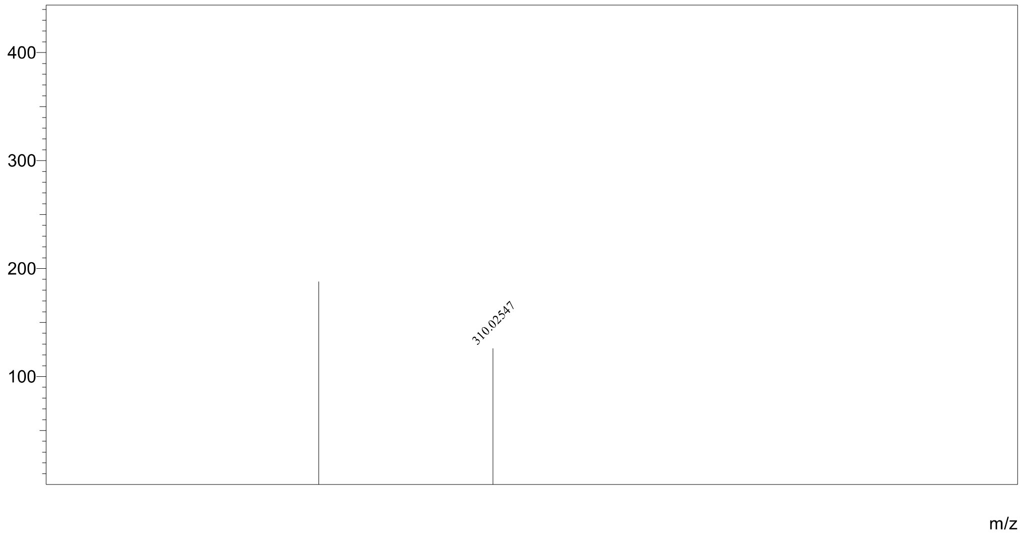

l-PheBx mass spectrum

<Spectrum>

Line#:1 R.Time:----(Scan#:----)
MassPeaks:1390
RawMode:Averaged 0.710-0.713(427-429) BasePeak:239.22514(270571)
BG Mode:Calc Segment 1 - Event 1

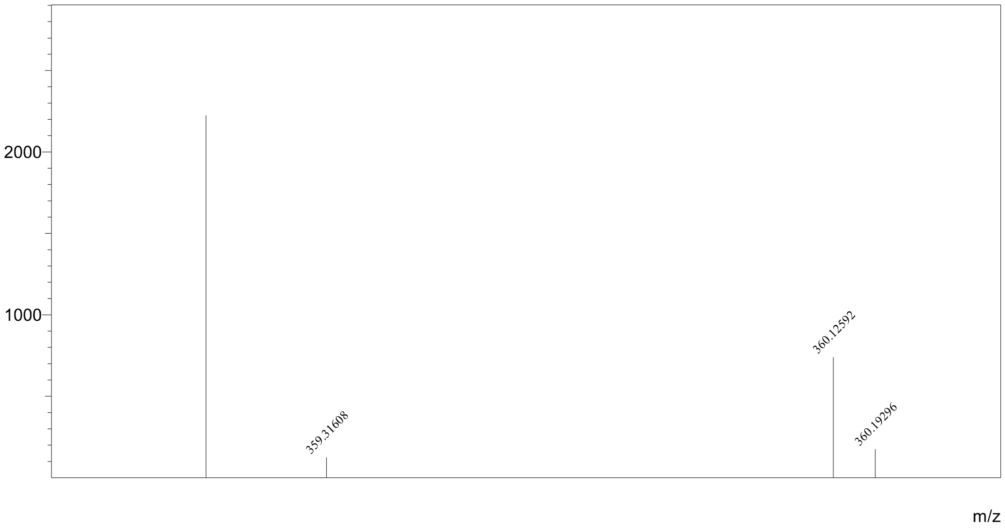

l-AlaBx mass spectrum

<Spectrum>

Line#:1 R.Time:----(Scan#:----)
MassPeaks:2557
RawMode:Averaged 0.698-0.702(420-422) BasePeak:102.12757(78905)
BG Mode:Calc Segment 1 - Event 1

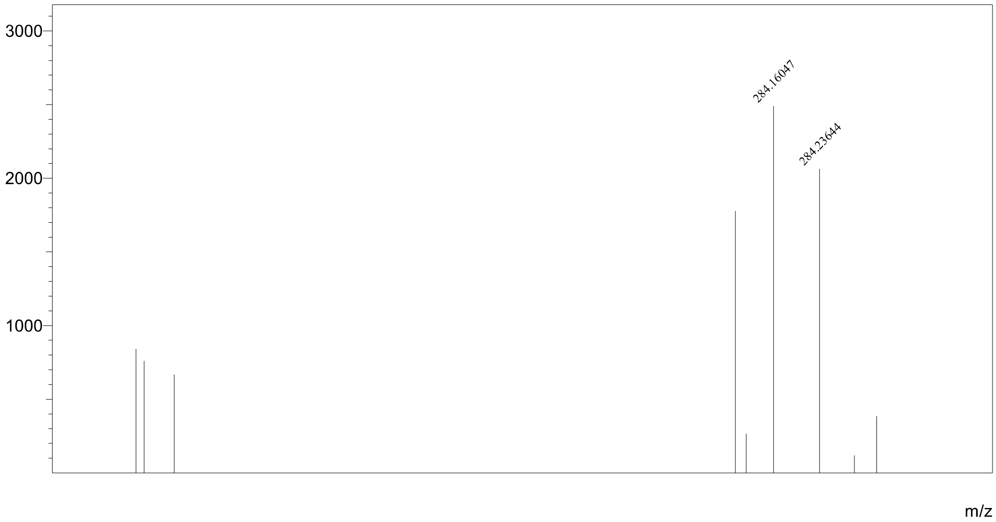

Supplement: Supplementary file 1 [file molecules-29-02116-s001.zip › molecules-2972696-supplementary.pdf]
